# Internet Use Patterns Among Korean Baby Boomers: Factors Influencing Gender Differences

**DOI:** 10.1093/geroni/igaf122.2807

**Published:** 2025-12-31

**Authors:** Jeehoon Kim, Ji Hyun Lee, Hee Lee, Young Ji Yoon, Dongwook Kim

**Affiliations:** Idaho State University, Pocatello, Idaho, United States; Montana State University, Bozeman, Montana, United States; The University of Alabama, Tuscaloosa, Alabama, United States; Colorado State University Pueblo, Pueblo, Colorado, United States; Arizona State University, Phoenix, Arizona, United States

## Abstract

Korean Baby Boomers (born between 1955 and 1963) are entering older adulthood with unprecedented educational and economic resources, enabling them to navigate the digital landscape more proficiently than previous generations. However, little is known about the patterns of Internet use and the gender-specific factors influencing their use. Using the 2012 Korean Baby Boomers Panel Study data, latent class analysis was conducted with eleven online activities. Multinomial logistic regression analysis was performed (reference: rare users) separately by gender. Predictors included health, social network size, social relationships, activity participation, loneliness, attitudes toward Internet use, and demographic variables. The participants (n = 3,227, Age M(SD)=52.8(2.6), 54% female) responded. Three use patterns were identified: rare users (46.5%), moderate users (43.9%), and maximizers (9.6%). Men were more likely to be moderate users or maximizers than women. Compared to rare users, both moderate users and maximizers engaged more in social activities, had more positive attitudes toward the Internet, higher educational attainment, and were younger. For women, moderate users and maximizers met friends/neighbors more often than rare users. Moderate users had smaller social networks, while maximizers were more likely to be single than rare users. For men, moderate users met friends/neighbors more often, while maximizers met relatives more often. Male maximizers were more likely to feel lonely than rare users. All findings had p-values less than .05. The gender differences in the relationship between social relationships, social networks, loneliness, and Internet use patterns will be discussed, along with intervention strategies for alleviating loneliness and enhancing social inclusion for maximizers.

